# Binahong (*Anredera cordifolia* (Tenore) Steen.) Leaf Extract Modulates Fatty Acids and Amino Acids to Lower Blood Glucose in High-Fat Diet-Induced Diabetes Mellitus Rats

**DOI:** 10.1155/2021/8869571

**Published:** 2021-04-28

**Authors:** Dwitiyanti Dwitiyanti, Yahdiana Harahap, Berna Elya, Anton Bahtiar

**Affiliations:** ^1^Universitas Indonesia, Kampus UI, Depok 16424, West Java, Indonesia; ^2^Department of Bioanalysis, Universitas Indonesia, Kampus UI, Depok 16424, West Java, Indonesia; ^3^Department of Phytochemistry, Universitas Indonesia, Kampus UI, Depok 16424, West Java, Indonesia; ^4^Department of Pharmacology and Toxicology, Universitas Indonesia, Kampus UI, Depok 16424, West Java, Indonesia

## Abstract

Patients with diabetes are 1.6 times more likely to use complementary alternative medicine than nondiabetic patients. Previous studies have shown that *Anredera cordifolia* (Tenore) Steen. (*A. cordifolia*) leaf extract has the capacity to lower blood glucose, but the actual mechanisms are unclear. Therefore, in this study, we explored the effect of *A. cordifolia* leaf extract on the metabolism of fatty acids and amino acids. Six-week-old male Wistar rats were randomly divided into six experimental groups (*n* = 5 per group). Two groups were fed with a regular diet or a high-fat diet (HFD) for six weeks. The regular diet and HFD groups were administered with 0.5% carboxymethylcellulose as a vehicle, and HFD rats were also fed with a suspension of glibenclamide (0.51 mg/kg body weight (BW)) or *A. cordifolia* leaf extract (25, 50, and 100 mg/kg BW). During the whole treatment, BW and food intake were recorded weekly. The rats were euthanized seven weeks after treatment. Blood glucose was evaluated by spectrophotometry, while fatty acids and amino acids were evaluated using a gas chromatography/flame ionization detector (GC/FID). All doses of *A. cordifolia* administration reduced blood glucose significantly, and 50 mg/kg BW was most effective in lowering blood glucose, similar to the effects of glibenclamide. *A. cordifolia* leaf extract affected the levels of medium-chain fatty acids, especially at 50 mg/kg BW. In contrast, glibenclamide affected long-chain fatty acids (LCFAs) to lower blood glucose. Based on the analysis conducted, we conclude that administration of *A. cordifolia* leaf extract can decrease blood glucose levels by regulating fatty acid metabolism and that a dose of 50 mg/kg BW in rats was the optimal dose.

## 1. Introduction

Diabetes mellitus is characterized by chronic hyperglycemia resulting from defects in insulin secretion, insulin action, or both. Diabetes mellitus is also associated with an increased risk of microvascular and macrovascular diseases [1].

The metabolism of foodstuffs is an altered type I and type II diabetes mellitus. The efficiency of glucose uptake and the utilization by most body cells is prevented by the lack of insulin or insulin resistance. Therefore, blood glucose concentration increases, the cell utilization of glucose decreases, and the utilization of fats and proteins also increases. The concentrations of branched-chain amino acids (BCAAs) in plasma are elevated in human and animal models of obesity [2]. BCAAs are hypothesized to be responsible for some beneficial effects of high-protein diets, including improving body weight (BW) control and adiposity. BCAAs improve muscle glucose uptake, whole-body glucose metabolism, and oxidation. BCAAs in plasma stop increasing due to a block in mitochondrial branched-chain amino acid aminotransferase, which has also been associated with improvements in glucose tolerance and resistance to diet-induced obesity [2].

Free fatty acids (FFAs) are essential for the normal function of pancreatic *ß* cells. These cells have the capacity to reverse insulin resistance, and their failure results in type II diabetes [3]. Elevated FFA can lead to the accumulation of fat depots in muscle, liver, and pancreatic *ß* cells, and accumulated triglycerides might promote a lipid environment that could interfere with metabolic signaling or action or both in these different tissues [4].

Recently, hyperlipidemia has been shown using metabolomics to be induced by high-fat diet- (HFD-) related metabolites, such as fatty acids, amino acids, phospholipids, and bile acids involved in fatty acid biosynthesis, beta-oxidation, amino acid metabolism, glycolysis, the TCA cycle, purine metabolism, energy metabolism, and bile secretion. These molecules have been demonstrated to be disturbed in metabolism in both animal models and humans [5].

The ideal treatment of type II diabetes should reverse insulin resistance and *ß*-cell dysfunction in treated patients and prevent, delay, or reverse long-term complications. Current treatment strategies are aimed at the amelioration of insulin resistance (diet, exercise, weight loss, and metformin and troglitazone therapy), augmentation of insulin supply (sulfonylurea and insulin therapy), or limitation of postprandial hyperglycemia (acarbose therapy). Future therapies may target (1) insulin resistance, using a multifaceted approach; (2) hepatic glucose production, using gluconeogenesis inhibitors; (3) excess nonesterified fatty acid production, using lipolysis inhibitors; and (4) fat oxidation, using carnitine palmitoyltransferase I and II inhibitors [6].

Many patients commonly use complementary alternative medicine as an alternative or in addition to their current medication regimen to prevent or treat diseases (7). Patients with diabetes have been found to be 1.6 times more likely to use complementary alternative medicine than other patients [7].

Some native tribes in Indonesia use the leaves of the *A. cordifolia* (*Anredera cordifolia* (Tenore) Steen.) plant to empirically reduce blood sugar, even as vegetables [8]. Other studies report that *A. cordifolia* leaf extract can inhibit *α*-glucosidase with an IC_50_ value of 54.24 *μ*g/ml. *A. cordifolia* leaf methanol extract at a dose of 50 mg/kg body weight (BW) and 200 mg/kg BW reduced blood glucose levels of alloxan-treated mice after seven days by 61.02% and 60.68%, respectively, while after 14 days, the decrease in glucose levels reached 75.64% and 66.61%, respectively, and was histologically shown to repair *β*-pancreatic cell damage.

In our previous study, we characterized 95% ethanolic *A. cordifolia* extract as containing 1.35% flavonoids and 1.031% vitexin [9]. Vitexin is a C-glycosylated derivative of apigenin [10], is known to have antidiabetic properties, and showed the most potent PTP1B inhibitory activity [10]. There is, to our knowledge, no data concerning the effect of *A. cordifolia* leaf extract on the fatty acid and amino acid profiles in serum to evaluate alterations in metabolism. Therefore, it is necessary to research the role of *A. cordifolia* leaf extracts on fatty acid and amino acid metabolism to elucidate the mechanism by which it lowers the glucose concentration in plasma.

## 2. Materials and Methods

All chemicals are purchased from Merck or Sigma except when specifically mentioned. Plant identification was carried out at the Bogoriense Herbarium, Botany-Biology Research Center, LIPI Cibinong, Indonesia (certificate no. 2285/IPH.1.01/If.07/IX/2018). *A. cordifolia* leaves were obtained from the Scientific Tourism Area, Bogor Spice and Medicinal Research Institute (Balitro) Bogor, Indonesia.

### 2.1. Plant Extracts Preparation

Fresh *A. cordifolia* leaves were washed and air-dried for four days and then powdered. The powder obtained was extracted by maceration using 96% ethanol and then filtered using filter paper. The maceration results were evaporated using a rotary evaporator to obtain a concentrated extract that could still be poured [9].

### 2.2. Animals and Experimental Design

All animals in this experiment were used following the animal care guidelines issued by Muhammadiyah University, HAMKA (certificate no. 02/19.04/0183). Thirty male Wistar rats were purchased from an animal breeding company in Solo, Indonesia, and were acclimated for one week prior to the initiation of the experiment. Rats were caged in an air-conditioned room (relative humidity 45%–65%) under a 12 h light-dark cycle at 30°C ± 2°C and were given free access to food and tap water [11].

Six-week-old male Wistar rats, weighing 150–200 grams, were randomly divided into six experimental groups (*n* = 5 per group). Two groups were fed with a regular diet or HFD *ad libitum* for six weeks and were administered 0.5% carboxymethylcellulose as a vehicle. HFD-induced type II diabetes mellitus (HFD T2DM) rats in the remaining groups were administered a suspension of glibenclamide (0.51 mg/kg BW) or *A. cordifolia* leaf extract (25, 50, or 100 mg/kg BW). During the treatment, BW and food intake were recorded weekly. The rats were euthanized seven weeks after the beginning of the treatment [8].

### 2.3. Induction of the Hyperglycemia of Diabetes Mellitus by HFD Feeding

Hyperglycemia was induced by providing an HFD of 20 g/day/200 g rat BW. The HFD feeding of this study was performed for 49 days; the composition of HFD is shown in Table 1 [12].

### 2.4. Biochemical Analysis

Plasma levels of glucose, fatty acids, and amino acids were determined as previously described [13]. Blood samples were taken on day 0 (before treatment), day 28 (28 days after induction of HFD), and day 49 (21 days after drug administration). Blood samples were taken from the retroorbital sinus and collected in a microtube. The blood was centrifuged at 7,000 rpm for 15 min, and the supernatant was used to obtain plasma samples. Plasma samples were analyzed to determine the levels of glucose and FFA.

### 2.5. Measurement of Glucose Levels

Glucose levels were measured by enzymatic colorimetry on a UV-VIS spectrophotometer at 500 nm using a glucose liquicolor kit. The absorbance obtained was compared with the absorbance of the blank control and then multiplied by the standard concentration [14].

### 2.6. Measurement of Fatty Acids and Amino Acids

Fatty acids and amino acids were measured using gas chromatography-flame ionization detection (GC-FID) (Alliance, Switzerland). Plasma FFAs were mixed with sulfonic acid, and 5 *µ*l was injected into the gas chromatograph-flame ionization detector [125 Α, 5 µm × 4.6 mm × 150 mm GC column (Xterra C8)] and analyzed [15].

### 2.7. Oral Glucose Tolerance Test (OGTT)

The OGTT was performed during the last week of the experiment. Rats have fasted for 12 h before OGTT. Subsequently, a 20% glucose solution (2 g/kg BW) was administered to the rats, blood samples were collected after 30, 60, and 120 min with heparinized capillary tubes, and blood samples were used to determine the glucose concentrations as described above [16].

### 2.8. Statistical Analysis

Data were expressed as means ± standard deviation. Data were statistically processed using SPSS version 16.

## 3. Results

### 3.1. Effects of HFD and *A. cordifolia* Leaf Extract on BW

Hyperglycemia was induced by providing an HFD of 20 g/day/200 g rat BW. The HFD induction of this study was performed for 49 days. The BW gain was the highest in the negative control group (negative), as shown in Table 2.

Obesity is associated with nonalcoholic fatty liver disease, which leads to excessive lipid accumulation in hepatocytes, also called steatosis [17]. Our study showed a significant reduction in the HFD-increased liver weight of rats treated with glibenclamide or *A. cordifolia* leaf extract. This result agrees with previous studies that showed that HFD-increased liver weight [18, 19].

### 3.2. Effects of HFD and *A. cordifolia* Leaf Extract on Blood Glucose and OGTT

Increased serum glucose was observed in HFD rats compared with the normal diet group (Table 3). Generally, rats displaying glucose levels more than 200 mg/dl were considered hyperglycemic [20]. In this experiment, glucose levels typically reached above 200 mg/dl after 21 days of HFD and were stable after 28 days HFD for an additional 28 days. Therefore, we administered glibenclamide and *A. cordifolia* leaf extract after 28 days of HFD treatment. Glucose levels declined below 200 mg/dl shortly after glibenclamide or *A. cordifolia* leaf extract administration and became insignificant from the normal group after 28 days of treatment. *A. cordifolia* leaf extract and glibenclamide showed significantly reduced serum glucose levels compared with the HFD group (*p* < 0.05).

**Figure 1:**
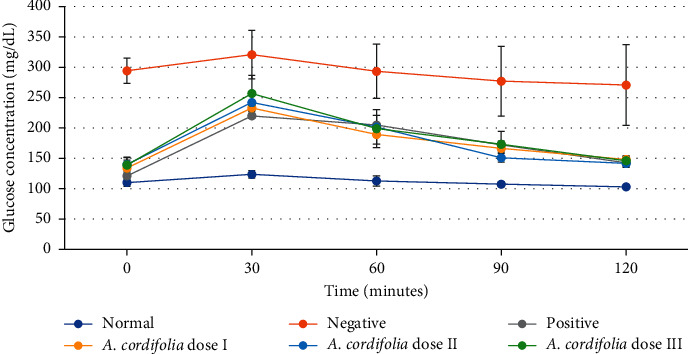
Oral glucose tolerance test. The administration of glucose leads in high blood glucose of diabetes mellitus model rats (negative control groups) at 0 times to 120 minutes after administration. The glibenclamide-treated rat (positive groups) and three doses of *A. cordifolia* leaf extract-treated rats could decrease the concentration of blood glucose after 30 minutes of glucose administration. The glucose level in the blood is expressed in mg/dl. The values indicate means ± SD of five rats per group.

**Figure 2:**
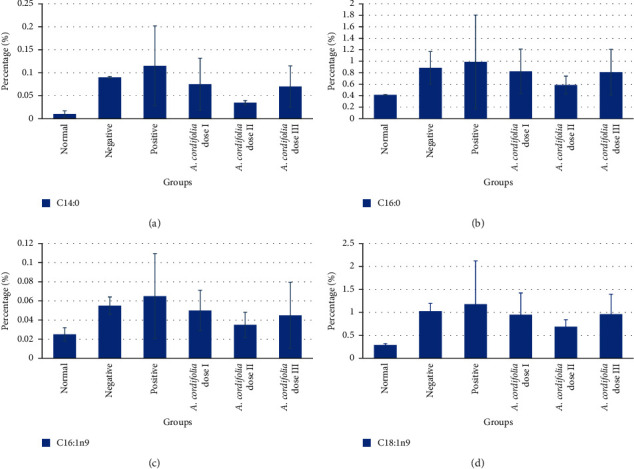
The level of plasma saturated fatty acid medium-chain (*C* < 20) higher in diabetic rats as compared to normal rats but lower in unsaturated fatty acids medium-chain. Oral administration of *A. cordifolia* leaves extract doses 25, 50, and 100 mg/kg body weight, and glibenclamide 0.51 mg/kg BW lowered the plasma fatty acid as compared to untreated diabetic rats. All values are described as mean ± SD for five rats in each group. (a) C14 : 0 = myristic acid methyl ester; (b) C16:0 = palmitic acid methyl ester; (c) C16:1n9 = palmitoleic acid methyl ester; (d) C18:1n9 = oleic acid methyl ester.

Treatment of HFD rats with *A. cordifolia* leaf extract significantly improved glucose tolerance (Figure 1). In the HFD group, *A. cordifolia* leaf extract-treated rats had reduced blood glucose area under the curve compared with the controls during the seven weeks on HFD (Figure 1). The blood glucose responses of rats on the HFD + *A. cordifolia* leaf extract were similar to those of the glibenclamide-treated rats (Figure 2). Similar results were observed when the rats were treated with oral glucose in other studies [21, 22]. The World Health Organization (WHO) recommends that GTT be used as a diagnostic method to classify people with reduced glucose tolerance. Nayak et al. showed a similar result: when normal Wistar rats were given oral glucose (2 g/kg), they showed no signs of intolerance compared to diabetic rats AUC after a 120-minute glucose challenge, and both test compounds and standards showed a reduction in blood glucose levels [23].

### 3.3. Effects of HFD and *A. cordifolia* Leaf Extracts on Fatty Acid Concentration

The profiles of fatty acids in HFD rats were detected by GC-FID. Sixteen fatty acids were detected, as shown in Figure 2. The HFD-increased almost all fatty acids, while *A. cordifolia* leaf extract reduced the increases in medium-chain fatty acids such as oleic acid (C16 : 0) and palmitic acid (C18 : 0), whereas glibenclamide reduced the increases in long-chain fatty acids, especially behenic acid (C22 : 0).

### 3.4. Effects of HFD and *A. cordifolia* Leaf Extract on Amino Acid Concentration

**Figure 3:**
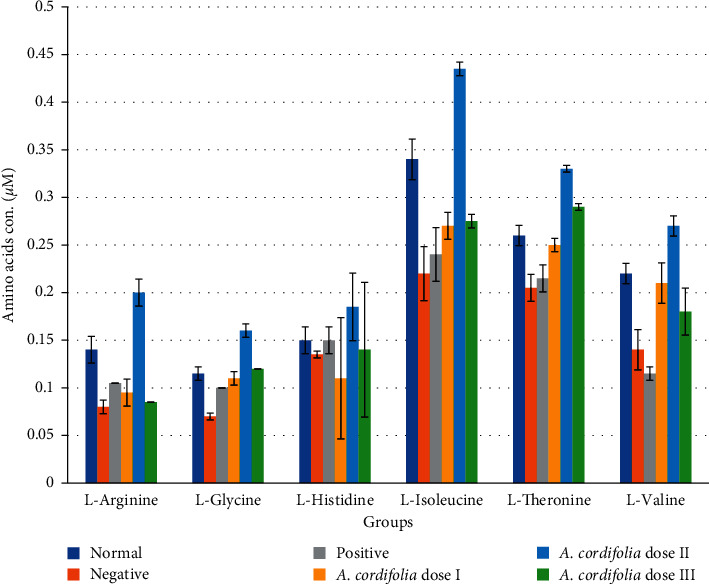
The level of plasma-amino acids lowers in diabetic rats as compared to normal rats. HFD lowered most of the amino acids, oral administration of *A. cordifolia* leaves extract doses 25, 50, and 100 mg/kg body weight, and glibenclamide 0.51 mg/kg BW increased the plasma-amino acid as compared to untreated diabetic rats. All values are described as mean ± SD for five rats in each group.

From the profiles of amino acids following HFD, all 20 amino acids were evaluated (Figure 3). HFD reduced the levels of almost all amino acids, while *A. cordifolia* administration, especially dose II (50 mg/kg BW), increased amino acid concentrations of L-arginine, L-isoleucine, L-valine, and L-glycine. Glibenclamide administration did not alter the levels of these amino acids, indicating that these compounds worked via different mechanisms to reduce plasma glucose.

## 4. Discussion

The current research was intended to investigate the effect of *A. cordifolia* leaf extract on HFD-induced diabetic Wistar rats. The administration of HFD in Wistar rats increased BW by approximately 25.60 g, similar to results reported in previous studies [23–25]. The composition of HFD was mainly fructose and butter as sources of saturated fatty acids (SFAs). Fructose entry into the liver is broken down into dihydroxyacetone phosphate and glyceraldehyde-3-phosphate. Dihydroxyacetone phosphate ultimately becomes the glycerol backbone of triglycerides, and glyceraldehyde-3-phosphate becomes the acetyl-CoA moiety required for *de novo* lipogenesis and the synthesis of fatty acids [26]. The postprandial insulin levels are more significant following the addition of saturated fat (butter) [27]; therefore, in this study, high glucose concentrations were used as a marker for diabetes mellitus.

HFD can induce nonalcoholic fatty acid liver disease (NAFLD) due to an imbalance between energy intake and expenditure. Leptin and its receptor LepR regulate BW by balancing food intake and energy expenditure by activating AMPK. AMPK is a major energy sensor of the cell and regulates hepatic and adipose lipid metabolism by modulating lipogenesis, lipolysis, gluconeogenesis, and adipogenesis [28].

HFD destroy the equilibrium between the formation and degradation of lipids and lead to excessive lipid deposition in hepatocytes, resulting in hepatic steatosis, NAFLD, or even more harmful conditions such as fibrosis or cirrhosis [29]. Elevated serum levels of total cholesterol (TC) and low-density lipoprotein (LDL)-c are important risk factors for developing atherosclerosis [30]. In our study, *A. cordifolia* leaf extract significantly reduced the serum lipid profile, indicating that it may be helpful in suppressing the formation of atherosclerosis.

Monounsaturated and polyunsaturated fatty acids showed positive correlations with high-density lipoprotein and negative correlations with TC, triglycerides, and LDL levels. In HFD, various tested SFAs and the total SFA increased due to the high-fat concentration, which contains abundant SFAs, especially C16 : 0 and C18 : 0 [31]. Studies that focused on lipid peroxidation in pathological conditions have shown that high levels of unsaturated fatty acids in cells produce reactive oxygen species and result in membrane and mitochondrial permeability, leading to cell apoptosis and death [32, 33]. C18 : 1 and C20 : 1 are monounsaturated fatty acids (MUFA), the accumulation of which induces the expression of Bim and FasL, molecules involved in apoptosis, as well as enhancing endoplasmic reticulum stress, resulting in hepatocellular damage [34]. Oleic acid (C18 : 1) is the predominant MUFA induced by HFD. Its elevation is speculated to be caused by the increase in Δ9 desaturases [35]. The activity of Δ9 desaturases is estimated using the ratio of C18 : 1/C18 : 0 and has been shown to be increased in both the serum and liver following HFD. Δ9 desaturase is also known as stearyl-coenzyme desaturase 1 (SCD1). This enzyme can catalyze the transformation from stearyl-coenzyme A (C18 : 0) to oleoyl-coenzyme A (C18 : 1). When comparing normal and *SCD1*-knockout mice fed HFD, insulin sensitivity was enhanced, and lipid accumulation in the liver was reduced in the *SCD1*-knockout group [32], indicating that the deletion of Δ9 desaturase could help reduce insulin resistance and hepatic steatosis. Furthermore, HFD has also been shown to contribute to the activation of Δ9 desaturase and results in the generation of C18 : 1. The correlation between serum C18 : 1 and liver C18 : 1 concentrations and hepatic steatosis, inflammation, and cell ballooning scores in our previous experiments suggested that the metabolic change in C18 : 1 in mouse sera might reflect liver damage related to HFD [33].

In this study, decreased levels of alanine and certain glucogenic amino acids (e.g., isoleucine and valine) and elevated levels of glucose in HFD-fed rats implied that glycolysis and aerobic oxidation of glucose were inhibited and that energy consumption might be shifted toward lipid oxidation in response to hyperlipidemia. This was also observed in apolipoprotein E knockout mice suffering similar energy metabolism impairment [34, 35].

The concentration of serum lipids is elevated in diabetes, which is a high-risk factor for coronary heart disease. Under reasonable conditions, insulin activates the enzyme lipoprotein lipase, which hydrolyzes triglycerides. However, in a diabetic state, lipoprotein lipase is not activated or is insufficiently expressed due to insulin deficiency, resulting in hypertriglyceridemia [36].

In metabolic syndrome patients, in the absence of glucose, insulin homeostasis disturbances and insulin resistance led to high amounts of polyunsaturated fatty acids (18 : 2 n6, 18 : 3 n3, 22 : 4 n6) and lower concentrations of SFA (12 : 0, 14 : 0, 16 : 0, 17 : 0) in plasma. A low concentration of polyunsaturated fatty acids (18 : 3 n3, 20 : 4 n6) with a predominance of SFA (14 : 0, 18 : 0) was also reported. In metabolic syndrome patients, regardless of the carbohydrate metabolism status, high levels of leukotriene B4 and 6-keto-prostaglandin-F1α in serum have been found [37].

Insulin deficiency produced complex alterations in the concentrations of amino acids in the plasma and heart muscle; the concentrations of some amino acids (alanine, valine, leucine, and isoleucine) increased, while others were decreased and a small number were unchanged [38].

## 5. Conclusions

Based on the analyses conducted, it can be concluded that the administration of *A. cordifolia* leaf extract decreased glucose levels in HFD T2DM rats by regulating fatty acid and amino acid metabolism. A dose of 50 mg/kg BW *A. cordifolia* leaf extract elicited a better effect than other dosages or glibenclamide.

## Figures and Tables

**Table 1:** The composition of a regular diet and HFD per 10 g diet.

	Regular diet (g)	High-fat diet (g)
Protein	1.20	1.50
Fat	0.40	1.20
Carbohydrate	0.70	3.50

**Table 2:** Bodyweight and liver weight of rats.

Group	Initial body weight (g)	Final body weight (g)	Weight gain (g)	Liver weight (g)	Liver weight-body weight ratio
Normal	256.80 ± 21.98	262.80 ± 19.61	6.00	9.75 ± 0.29	26.9
Negative	200.40 ± 30.79	225.80 ± 30.21	25.40*∗*	11.62 ± 0.39*∗*	19.4
Positive	206.00 ± 45.84	199.40 ± 42.40	−6.60#	9.39 ± 0.39#	21.2
*A. cordifolia* dose I	204.00 ± 23.37	223.00 ± 13.64	19.00	9.54 ± 0.49#	23.3
*A. cordifolia* dose II	226.20 ± 35.20	225.60 ± 32.20	−0.60#	8.75 ± 0.72#	25.7
*A. cordifolia* dose III	194.60 ± 31.35	190.00 ± 12.31	−4.60#	9.62 ± 0.42#	19.7

*∗*Significantly different with normal group (*p* < 0.05). #Significantly different with the negative group (*p* < 0.05).

**Table 3:** Levels of glucose in experimental rats after 21, 28, 35, 42, and 49 days of treatment.

Groups	Day 0	Day 21	Day 28	Day 35	Day 42	Day 49
Normal	89.86 ± 5.58	93.57 ± 3.87	97.29 ± 2.81	101.00 ± 5.23	104.00 ± 6.63	110.57 ± 6.05
Negative	86.29 8.18	226.29 ± 26.44*∗*	276.43 ± 29.26*∗*	287.14 ± 18.11*∗*	283.00 ± 14.06*∗*	292.71 ± 18.06*∗*
Positive	83.86 ± 8.99	208.71 ± 12.23*∗*	250.14 ± 20.76*∗*	175.57 ± 12.08*∗*	132.14 ± 25.54*∗*	121.14 ± 12.51#
*A. cordifolia* dose I	88.71 ± 7.16	223.43 ± 7.02*∗*	265.14 ± 15.59*∗*	178.71 ± 8.43*∗*	151.86 ± 11.04*∗*	134.14 ± 7.43#
*A. cordifolia* dose II	84.14 ± 6.20	210.14 ± 12.79*∗*	257.43 ± 32.06*∗*	169.29 ± 18.82*∗*	166.71 ± 11.13*∗*	142.86 ± 14.11*∗*
*A. cordifolia* dose III	84.43 ± 11.98	211.43 ± 17.65*∗*	255.29 ± 26.95*∗*	183.14 ± 18.34*∗*	148.14 ± 16.55*∗*	135.71 ± 12.18*∗*

*∗*Significantly different with the normal group (*p* < 0.05). #Significantly different with the negative group (*p* < 0.05).

## Data Availability

The data that support the findings of this study are available from the corresponding author (AB) upon reasonable request.

## Abbreviations

BCAAs: Branched-chain amino acids
BW: Body weight
FFAs: Free fatty acids (FFAs)
HFD: High-fat diet (HFD)
TCA cycle: Tricarboxylic acid cycle
PTP1B: Protein tyrosine phosphatase 1B
UHAMKA: Muhammadiyah University, HAMKA
HFD T2DM: HFD-induced type II diabetes mellitus
UV-VIS: Ultraviolet-visible
GC-FID: Gas chromatography-flame ionization detection
OGTT: Oral glucose tolerance test
SPSS: Statistical Package for the Social Sciences
SFAs: Saturated fatty acids
NAFLD: Nonalcoholic fatty acid liver disease
AMPK: AMP-activated protein kinase.
LDL: Low-density lipoprotein
TC: Total cholesterol
MUFA: Monounsaturated fatty acids
SCD1: Stearyl-coenzyme desaturase 1.
